# Potential explanation of limb combination performance differences for two-limb coordination tasks

**DOI:** 10.14814/phy2.12301

**Published:** 2015-02-23

**Authors:** Kento Nakagawa, Tetsuro Muraoka, Kazuyuki Kanosue

**Affiliations:** 1 Faculty of Sport Sciences, Waseda University2-579-15 Mikajima, Tokorozawa, Saitama, 359-1192, Japan; 2 Japan Society for the Promotion of ScienceChiyoda-Ku, Japan; 3 College of Economics, Nihon University1-3-2 Misaki-Cho, Chiyoda-Ku, Tokyo, 101-8360, Japan

**Keywords:** Closed-loop control, interlimb coordination, kinesthetic tracking, limb combination

## Abstract

Rhythmic two-limb coordinated movements in the sagittal plane are variable and inaccurate when the movements are in the opposite direction as compared with those in the same direction (directional constraint). The magnitude of directional constraint depends on the particular limb combination. It is prominent in ipsilateral hand-foot coordination, but minimal in bimanual hand coordination. The reason for such differences remains unclear. In this study, we investigated the possible mechanisms underlying the production of the difference that depend on limb combination. Subjects performed two-limb rhythmic coordinated movements either in the same or in the opposite direction for three separate limb combinations (bilateral hands, contralateral hand and foot, and ipsilateral hand and foot). For each combination two different tasks were performed. In the first condition, subjects actively moved two limbs (active condition). Second, subjects actively moved one limb in coordination with a passively moved limb (passive condition). In the active condition, the directional constraint was dependent upon the limb combination, as reported in previous studies; the directional constraint was quite prominent in ipsilateral combinations, intermediate in contralateral combinations, and minimal for bilateral combination. However, differences in the directional constraint did not depend on limb combination for any combination in the passive conditions which apparently utilized closed-loop control. In other word, the difference depending on limb combination disappeared when control strategies become uniformly closed-loop. Thus, we speculate that the control strategy utilized depends on limb combination in the active condition. Additionally, different mechanisms other than closed-loop control also would have influence depending on the particular limb combination. This may result in differences in performance depending upon the limb combination.

## Introduction

There are many situations that require coordinate multi-limb movements. These situations occur not only in the occurrences of daily life, but also in numerous activities such as participating in sports and playing musical instruments. Specific constraints characterized the different multi-limb coordinations (Swinnen 2002). For example, when subjects try to move the ipsilateral hand and foot in the sagittal plane, opposite directional movements are more variable, and incorrect responses occur more frequently than with same directional movements (*directional constraint*) (Baldissera et al. 1982; Carson et al. 1995; Muraoka et al. 2013). With an increase in movement frequency, opposite directional movements tend to shift to same directional movements. Some studies have suggested that the magnitude of the directional constraint depends upon the particular “limb combination” (Kelso and Jeka 1992; Swinnen et al. 1995, 1997; Serrien et al. 2000; Hiraga et al. 2004, 2005; Meesen et al. 2006). While directional constraint is prominent when the subjects move ipsilateral upper and lower limbs (e.g., right hand and right foot: “*ipsilateral*” combination), the constraint is less prominent when moving an upper limb and a contralateral lower limb (e.g., right hand and left foot: “*contralateral*” combination). Furthermore, for coordinated movements of “*bilateral*” limbs (e.g., both hands or both feet) in the sagittal plane there is little or no directional constraint (Riek and Carson 2001). Such differences in the extent of the directional constraint that depend upon the particular two-limb combination mainly derive from disparities in the variability and accuracy of opposite directional movements. Opposite directional movements are the most variable and inaccurate for ipsilateral combinations. They are more stable and accurate for contralateral combination. And they are particularly stable and accurate for bilateral combination. However, same directional movements are stable and accurate in any limb combination. In this study, we denote differences in the extent of directional constraint that depend on limb combination as “*combination effect*”.

Although combination effects have been well recognized, the underlying mechanism is not well understood. It has been suggested that this effect derives from a difference in biophysical properties among effectors (Kelso and Jeka 1992). For example, the moments of inertia, lengths or eigenfrequencies must be different, especially between the upper and lower limbs. Serrien and Swinnen (1998) investigated whether differences in these properties produce the combination effect using a model involving both (bilateral) arms, as well as contralateral and ipsilateral arms and legs. They added weight to a single limb to change the inertial characteristics. This was carried out to rend the arms on both sides dissimilar, or to make the arm and leg similar. Although the performance deteriorated under all combinations, the combination effect was substantially preserved. Therefore, they rejected the view that inertial differences produce the combination effect (Serrien and Swinnen 1998). Thus, the origin of the combination effect still remains an open question.

Recently, our group compared the coordination of actively moved ipsilateral hand and foot (active condition) with an active hand movement in coordination with a passive movement of the ipsilateral foot (passive condition) (Nakagawa et al. 2013). Interestingly, directional constraint was preserved even in the passive condition in which the subjects moved only one limb. However, directional constraint disappeared when a hand was actively moved without coordination with the passively moved foot (nontracking condition). The substantial difference between the passive and nontracking conditions is that a process of “error correction” with a closed-loop control should work in the former and not in the latter. Thus, it was concluded that the directional constraint derived at least partly from a process of “error correction” that is accomplished with a closed-loop control system. On the other hand, it has been suggested that closed-loop control is not important for directional constraint in bimanual coordination in a horizontal plane (Ridderikhoff et al. 2005). Thus, control strategy might depend on the particular limb combination. For “stable” opposite directional movements of a bilateral combination, open-loop rather than closed-loop control might be utilized. From these considerations, we hypothesized that the combination effect appears because of a difference in the control strategy among the various limb combinations, and the combination effect should disappear in situations where the same control strategy is utilized among limb combinations. The objective of this study was to investigate this hypothesis. In accordance with previous studies, we utilized the passive condition in which subjects had to move one limb with the use of kinesthetic tracking (closed-loop control) to follow the passive movements of another limb. The methodology we utilized is well established and has been utilized by a number of investigators to analyze the contribution of the projections of efferent or afferent signals, and interactions between them especially in two-limb coordination task (Stinear and Byblow 2001; Ridderikhoff et al. 2005; de Boer et al. 2011, 2012; Nakagawa et al. 2013). If the responses of the active and passive conditions differ, the control strategy of the active condition would not solely depend on closed-loop control, and other mechanisms of necessity would be involved. We utilized three limb combinations; (1) bilateral hands, (2) contralateral hand and foot, and (3) ipsilateral hand and foot.

## Methods

### Subjects

Eleven healthy right-handed adults (nine males and two females, 24 ± 2 years of age) participated in this experiment. Written informed consent was obtained from all subjects. The study was approved by the Human Research Ethics Committee of Waseda University (2010–107).

### Materials

**Figure 1:**
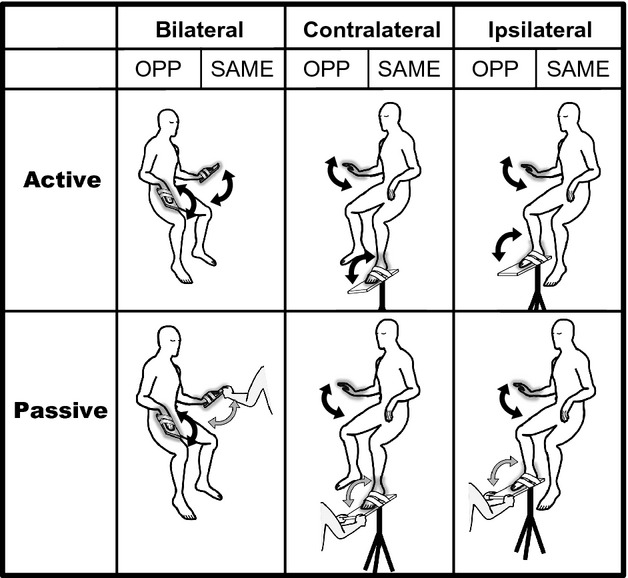
The experimental setup and tasks. Subjects performed two-limb coordinated movements under three combinations (bilateral, contralateral, ipsilateral). Each condition (active, passive) had two directions (ISO, the same direction; OPP, the opposite direction). Black arrows indicate active movements executed by the subject, whereas gray arrows indicate passive movements executed by the experimenter.

During the experiment, the subjects sat comfortably in a chair with the right forearm fixed in a prone position on an armrest. The sole of the right/left foot was taped to a wooden board attached to a tripod. This allowed the subject's foot to be moved in the sagittal plane for ipsilateral/contralateral combinations (Fig.1). For task involving bilateral combinations, the subjects sat in a different chair which contained armrests on both sides. In this case, wooden boards were attached to palm of the both hands (Fig.1). The angular displacements of each limb were measured at 1 kHz using electrical goniometers (SG150, Biometrics, Newport, UK). The joint angle signals were low-pass filtered with a cutoff frequency of 10 Hz. All signals were converted into a digital format with an A/D converter system (Power lab 16/30, ADInstruments, Nagoya, Japan), and stored on a computer.

### Task

During execution of the tasks, subjects kept their eyes closed and wore noise-cancelling headphones (ATH-ANC9, Audio-Technica, Tokyo, Japan). The tasks required rhythmic flexion and extension movements in the sagittal plane. There were two directions (the same direction and opposite direction), three limb combinations (ipsilateral, contralateral, and bilateral) and two conditions (active and passive). In the same (SAME) and opposite (OPP) directional movements, two limbs were moved in the sagittal plane in the same and opposite directions, respectively. The three limb combinations were: right hand and right foot (ipsilateral), right hand and left foot (contralateral), and right hand and left hand (bilateral). In the active condition subjects performed voluntary movements of two limbs at the pace indicated by the metronome sound, and in the passive condition subjects moved their right hand actively in coordination with the other limb which was passively moved by the experimenter. Each task consisted of five trials, resulting in a total of 60 trials (2 directions × 3 combinations × 2 conditions × 5 trials).

### Procedure

Before the experiment, subjects practiced moving two limbs to a pace set by a metronome which was set at the same beat (2 Hz) that was used during the experiment. Subjects executed 20 cyclical movements of two limbs. In the active task, subjects began the movement of their limbs arbitrarily. In the passive task, the experimenter, who heard the metronome sound via headphones moved the subject's second limb (ipsilateral: right foot, contralateral: left foot, bilateral: left hand). Subjects started movement of the right hand after perceiving a kinesthetic signal derived from passive movement of the second limb. They tried to relax the muscles of the second limb, and to not move that limb voluntarily. Each combination was carried out separately. The order of the four conditions (active_OPP, active_SAME, passive_OPP, passive_SAME) was randomized for each combination of limbs. The order of the combination block was also randomized across subjects. Subjects were allowed to rest *ad libitum* between sessions.

### Data analysis

To evaluate the performance of two-limb coordination, the relative phase (Φ) between the movements of the hand and foot was calculated for each cycle as Φ_*hf*_ = 360*°*(*t*_*f,i*_* − t*_*h,i*_)/(*t*_*f*,*i + 1*_* *−* t*_*f*,*i*_), where *t*_*h*,*i*_ and *t*_*f*,*i*_ indicate the time of the *i* th peak extension of the hand and foot, respectively (Carson et al. 1995; Ridderikhoff et al. 2005; Volman et al. 2006). To evaluate the variability and accuracy of the coordinated movements, standard of deviation (SDΦ) and absolute errors (AEΦ) of the relative phases between two limbs were used as indexes of variability and accuracy of two-limb coordination, respectively. SDΦ was standard deviation of relative phases in 1 trial (18 cycles). AEΦ was calculated by averaging the errors in 1 trial to the target relative phase (SAME: 0°, OPP: 180°), which was shown as the absolute value. We defined the degree of directional constraint in two different indexes as that obtained by calculating the difference between the performances of SAME and OPP ([AEΦ of OPP - AEΦ of SAME], and [SDΦ of OPP - SDΦ of SAME] as variability). Moreover, we calculated the difference between the peak extension and flexion angles in each cycle, and this was defined as movement amplitude for the hand and foot. The mean movement frequencies of each trial for hand and foot movements were also obtained. Each index was calculated with the data from the 1st to 18th cycles. Once a relative phase departed over 360° from the target relative phase, subsequent cycles were removed even though in some cases the relative phase returned to within 360°.

### Statistical analysis

The data based on relative phase (difference between SAME and OPP) were analyzed by a nonparametric Wilcoxon signed rank test, because the variances of each dataset were not equal (3 combinations (bilateral, contralateral, and ipsilateral) × 2 conditions (active, passive)) in each index (SDΦ and AEΦ).

We utilized a four-way ANOVA with repeated measures (limb (right hand, second limb) × combination × condition × direction) to examine the difference in movement frequency among tasks. When an interaction was found, a post hoc test (Tukey HSD) was performed. Although active movements of the right hand (first limb) commonly existed, the second limb differed depending on the combination. Movement amplitude in the second limb was analyzed by a separate approach. As different limbs (hand and foot) have different natural amplitudes, we did not compare the movement amplitude of the second limb of a combination. We performed two-way ANOVAs (condition × direction) for the ipsilateral and contralateral combination, and three-way ANOVAs (limb × condition × direction) for the bilateral hands combination. The level of significance for all tests was set to 0.05 in all analyses. Data values are expressed as mean ± standard error (SE).

## Results

### Relative phase distribution

**Figure 2:**
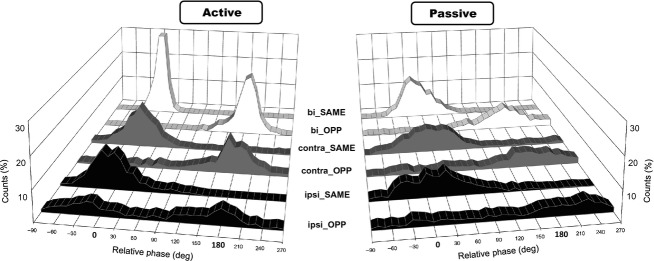
Histogram plots of proportion of the relative phase counts in each task. Left graph indicates the active condition, and right graph indicate the passive condition). Bilateral data are in the top two rows (white), contralateral in the middle rows (gray), and ipsilateral in the bottom rows (black).

Figure2 shows the distribution of relative phases for all subjects. In the active condition (left side), waveform of bilateral tasks (bilateral_OPP and bilateral_SAME) were similar and had very sharp peaks either at 0° (SAME) or at 180° (OPP). In the contralateral tasks, the waveforms also have peaks, but they are blunter than those of the bilateral tasks. However, the waveforms of the ipsilateral tasks prominently differed with movement direction. In ipsilateral tasks, while the peak appears at 0° for the SAME directional movement, two gentle peaks appeared in the OPP directional movement. This suggests that phase transition from the relative phases of 180° to 0° frequently occurred in the OPP directional movement. Interestingly, these differences in waveforms for the limb combinations were not so apparent in the passive conditions. Waveforms of the SAME tasks are similar among all limb combinations, with a peak at about 0°. On the contrary, waveforms of the OPP tasks have no distinct peak as do those of the SAME task. In other words, the active condition had a prominent “combination effect”, whereas the passive condition did not.

### Directional constraint

**Figure 3:**
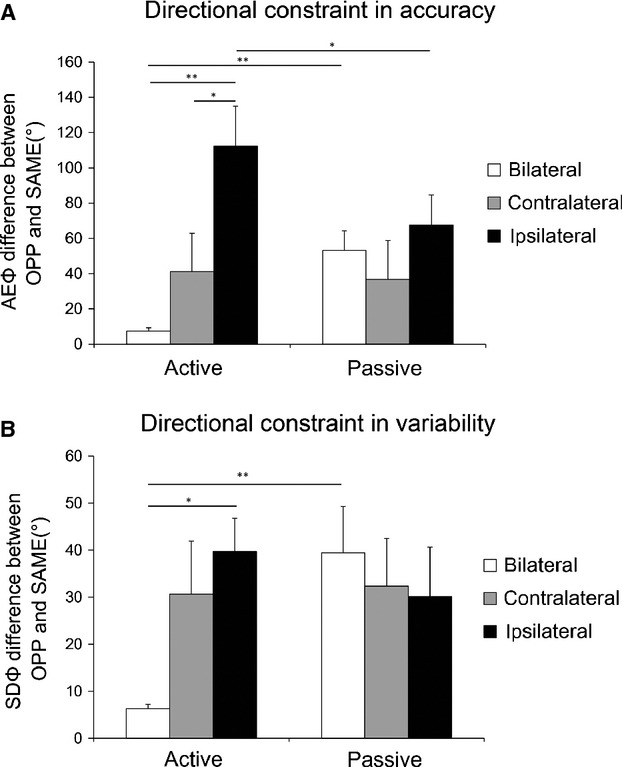
Difference in the directional constraint. The difference between the performances of OPP and SAME in accuracy (AEΦ) (A) and variability (SDΦ) (B) were shown as degrees of the directional constraint. Asterisks indicate significant differences detected by Wilcoxon signed rank test. In every graph, white bars indicate the data of bilateral, gray is contralateral, and black represents ipsilateral combination. Values are expressed as mean ± SE.

Figure3 shows the differences between the performance of SAME and OPP, which indicates the degree of directional constraint. In AEΦ there were significant differences between bilateral_active and ipsilateral_active (*P* < 0.01), contralateral_active and ipsilateral_active (*P* < 0.05), bilateral_active and bilateral_passive (*P* < 0.01), and ipsilateral_active and ipsilateral_passive (*P* < 0.05). In SDΦ, there were significant differences between bilateral_active and ipsilateral_active (*P* < 0.01), and between bilateral_active and bilateral_passive (*P* < 0.01). In the passive condition, there was no difference that depended upon the limb combination in either index.

### Movement frequency

A four-way ANOVA (limb × combination × condition × direction) detected a significant main effect of limb (right hand and second limb) (*F*_1,10_ = 10.55, *P* < 0.01), a main effect of direction (*F*_1,10_ = 24.87, *P* < 0.001), an interaction of limb × condition (*F*_1,10_ = 14.72, *P* < 0.01) and an interaction of limb × condition × direction (*F*_1,10_ = 10.90, *P* < 0.01). However, no significant difference between tasks was detected by the post hoc test (Tukey HSD).

### Movement amplitude

For movement amplitude of the hands in the bilateral combination, a three-way ANOVA detected a significant interaction of limb × condition (*F*_1,10_ = 6.33), *P* < 0.05) and interaction of condition × direction (*F*_1,10_ = 6.27, *P* < 0.05), but the post hoc test did not find significant differences between tasks. In the right hand for the contralateral combination, two-way ANOVA detected a significant main effect of direction (*F*_1,10_ = 6.10, *P* < 0.05). In cases of the left foot in the contralateral combinations, the right hand in the ipsilateral combination and the right foot in the ipsilateral combination, no significant main effect or interaction was detected.

## Discussion

The aim of this study was to investigate the mechanisms underlying the “combination effect” of two-limb coordinative movements. We focused on the possibility of a difference in control strategy rather than on structural differences between the limbs (Kelso and Jeka 1992). To this end, we tested the hypothesis that the difference depending on limb combination disappeared when control strategies become uniformly closed-loop. We compared performances in two-limb movements in the active condition (active movements of two limbs) and the passive condition (active movement of one limb in coordination with the passive movement of a second limb) for three different limb combination (bilateral hands, contralateral hand and foot, ipsilateral hand and foot). The relative phase distribution (Fig.2) and differences in the AEΦ and SDΦ between OPP and SAME tasks (Fig.3) showed the combination effect in the active condition. The order of degree of the directional constraint from the ipsilateral, contralateral to bilateral combination was observed in the active condition. This coincides with the results of previous studies (Kelso and Jeka 1992; Swinnen et al. 1995, 1997; Hiraga et al. 2004). On the other hand, no combination effect was observed in the passive condition. That is, no significant differences in the directional constraints in the passive condition were observed between limb combinations, in either accuracy or variability (Fig.3).

Frequencies and amplitudes of limb movements did not vary with tasks, either in the active or passive conditions, or with limb combinations. Only in the contralateral combination the amplitude of right hand (that was always moved actively) was bigger in the opposite directional movements than in the same directional movements for both the active and passive conditions. In addition, the difference in these amplitudes was only ∼7°, so it could not be a reasonable explanation for the observation that such a difference produced the “intermediate” directional constraint in the contralateral combination. Therefore, the movement frequency and amplitude would not likely be important factors in the production of the combination effect.

As noted above, a difference in directional constraint between the active and passive conditions appeared, and this was particularly prominent for the bilateral and ipsilateral combination. In the bilateral combination, while both same and opposite directional movements were almost perfectly performed in the active condition, the performance of the opposite directional movement deteriorated in the passive condition (Fig.2); that is, the directional constraint became prominent in the passive condition (Fig.3). What was the critical difference between the active and passive conditions of the bilateral combination? For one thing, subjects had to control the movements of both hands in the active condition, while they controlled only one hand in the passive condition. However, a difference in the number of hands controlled is not likely to be the reason why the directional constraint became prominent in the passive condition, because the directional constraint was greater rather than smaller in the passive condition.

In our previous study (Nakagawa et al. 2013), as well as in the study by Ridderikhoff et al. (2005), three factors (“interaction in efferent process”, “interaction of afferent signals”, and “error correction”) were proposed to underlie the directional constraint. As “interaction in efferent process” is defined as a function that works during the voluntary motion of two limbs without a contribution from afferent signals (Ridderikhoff et al. 2005; Nakagawa et al. 2013), such a function could not be involved with the directional constraint in the passive condition because the subjects only controlled the movements of one limb. In addition, there must be a difference in afferent signals between active and passive movements, because coactivation of *γ*-neurons exists in the former but not in the latter: That is, afferent signals should be stronger for actively moving limbs than for passively moving limbs. Then, “interaction of afferent signals”, if any, would be stronger in the active condition. However, this was not the case for the bilateral combination, in which the directional constraint was stronger in the passive condition. Thus, “interaction of afferent signals” might not the cause of the direction constraint at least for the bilateral combination. The critical factor that produced the directional constraint in the bilateral passive condition must have been “error correction”.

Error correction is an important aspect of closed-loop control, which apparently was operating in the passive condition. Thus, some alternative strategy must be working in the active condition, in which the directional constraint was not prominent for the bilateral combination (Fig.2). We speculate that the highest performance in the bilateral active condition would be accomplished using an “open-loop control”. In a similar situation, Riek and Carson (2001) found that for cyclic coordination of bilateral ankles in the sagittal plane there was no difference in performance between same and opposite directional movements. Indeed, movements of the lower limbs, including ankles, during walking (opposite directional movement) and hopping (same directional movement) are programmed so as to function with open-loop control (Zehr and Duysens 2004). Thus, it is quite probable that an open-loop system controls bimanual coordinated movements in the sagittal plane, similar to the control of bilateral ankle movements. In the passive condition, open-loop process cannot be brought into action, because the subject's task is to monitor kinesthetic information of both passively and actively moved limbs, calculate errors of the relative phases between them, and adjust them according to the instructions for the particular condition (OPP or SAME) with an exclusively closed-loop process (“error correction”).

If the process of “error correction” with a closed-loop control system were to produce a directional constraint in passive condition of bimanual coordination, the same mechanism should also work for hand-foot coordination in the active condition, which showed a prominent directional constraint (Fig.2, and 3), both in the ipsilateral or contralateral combination. Indeed, the previous studies have proposed that closed-loop control is mainly involved in ipsilateral hand-foot coordination (Baldissera et al. 1991; Swinnen et al. 1995; Nakagawa et al. 2013). However, other mechanisms might also contribute to the directional constraint in ipsilateral hand-foot coordination, because the directional constraint in the active condition was stronger than that in the passive condition. For example, the modulation of subliminal neural or electromyogram activation in the resting upper limb was observed during voluntary movement of the ipsilateral lower limb so as to enhance the same directional movement and to hinder the opposite directional movement (Baldissera et al. 2002; Baldissera and Esposti 2005; Byblow et al. 2007; McIntyre-Robinson and Byblow 2013). This diverged projection of efferent signals to nonworking muscles or muscles in the other limbs might be the cause of the enhancement of the directional constraint. However, the effects of this process, if any, would not be a necessary condition for the directional constraint, because phase transition from the opposite direction to the same direction also occurred in the subjects who did not show the diverged projection of efferent signals (McIntyre-Robinson and Byblow 2013), and the directional constraint in ipsilateral hand-foot coordination disappeared when a hand was actively moved without coordination with the passively moved foot (Nakagawa et al. 2013). Furthermore, Baldissera et al. (2006) proposed the possibility that the ipsilateral hand-foot coordination is accomplished utilizing a common rhythm generator without crossed feedback interaction between limbs, based on the data of neural or mechanical phase delay of the single- or two-limb movement. Thus, numerous mechanisms likely contribute to ipsilateral hand-foot coordination.

Then, how can we explain the difference in performance between the ipsilateral and contralateral hand-foot coordination? Likely there are other mechanisms that caused the directional constraint of the ipsilateral combination to be higher than that of the contralateral combination. One possibility is a difference in involvement of the motor cortices. The primary motor cortex on the side contralateral to the moving hand and foot is likely to be much more active for the ipsilateral combinations. On the other hand, the primary motor cortices of both sides would be expected to be working in the contralateral combinations. Thus, both hemispheres would share the load of controlling the two body parts, and thus performance might well be better in the contralateral combination than the ipsilateral combination. Alternatively, the difference in cortical inhibition between ipsilateral and contralateral hand-foot coordination has been confirmed (Fujiyama et al. 2009, 2012). This neural difference may have a relevance to the difference in the directional constraint. Furthermore, an inherent spinal neural circuit (pattern generator) may also contribute to the difference in the directional constraint between the ipsilateral and contralateral hand-foot combinations. Additional studies are needed to clarify the mechanisms involved in such of the differences.

The frequency of two-limb movement utilized in this study was 2 Hz. This is higher than that in previous studies (Kelso and Jeka 1992; Swinnen et al. 1995, 1997). Thus, the finding of this study may not apply to the other movement frequencies. In the active condition the combination effect was also observed in previous studies that utilized slower frequency (about 1 Hz). However, it is not known whether performance at these lower frequencies, or at frequencies above 2 Hz would show results similar to those of our study. Indeed, it has been proposed that the control strategy for bimanual coordinated movement in the horizontal plane changes depending on movement frequency (1 Hz to 3.5 Hz) (de Boer et al. 2011). Therefore, an investigation of the frequency dependence of the combination effect in the passive condition will be needed if conclusions of this study are to be generalized across viable frequencies.

In summary, for the purpose of this study we defined “combination effect” as differences in the extent of directional constraint that depend upon limb combination. Our hypothesis was that the combination effect depends on the difference in control strategy. For three of the limb combinations we utilized (bilateral hands or contralateral/ipsilateral hand and foot), we investigated movements in both the active and passive conditions. While a prominent combination effect was observed in the active condition, the combination effect disappeared in the passive condition, in which the same control strategy (closed-loop control) would likely be utilized for all limb combinations. It was argued, thus, that type of control depends on limb combination, and this may be the most important factor in the production of the combination effect.
